# Quality Assurance of Non-Invasive Prenatal Screening (NIPS) for Fetal Aneuploidy Using Positive Predictive Values as Outcome Measures

**DOI:** 10.3390/jcm8091311

**Published:** 2019-08-26

**Authors:** Wendy DiNonno, Zachary Demko, Kimberly Martin, Paul Billings, Melissa Egbert, Susan Zneimer, Dianne Keen-Kim, Peter Benn

**Affiliations:** 1 Natera, Inc., San Carlos, CA 95070, USA; 2 UConn Health, Farmington, CT 06030, USA

**Keywords:** non-invasive prenatal screening, cell-free DNA, aneuploidy, trisomy 21, positive predictive value, maternal age, quality assurance

## Abstract

Non-invasive prenatal screening (NIPS) based on the analysis of cell-free DNA in maternal plasma has been shown to have high sensitivity and specificity. We gathered follow-up information for pregnancies in women with test-positive NIPS results from 2014–2017 with quarterly assessments of positive predictive values (PPVs). A non-inferiority analysis with a minimum requirement of 70%/80% of expected performance for trisomy 21 and 18 was used to ensure testing met expectations. PPVs were evaluated in the context of changes in the population receiving testing. For all quarters, PPVs for trisomies 21 and 18 exceeded the requirement of > 70% of the reference PPV. Overall observed PPVs for trisomy 21, 18, 13 and monosomy X were similar for women aged <35 (90.9%, 95% Confidence Interval (CI) 88.6–92.7%) compared to women with advanced maternal age (94.5%, 95% CI 93.1–95.6%). Despite significant declines in test-positive rates from 1.18% to 0.62% for trisomy 21, and from 0.75% to 0.48% for trisomies 18, 13 and monosomy X combined, PPVs remained stable through the four-year interval. We conclude that quarterly evaluation of PPV provides an overview of past testing and helps demonstrate long-term consistency in test performance, even in the setting of increasing use by women with lower a priori risks.

## 1. Introduction

Non-invasive prenatal screening (NIPS) for fetal chromosomal abnormalities is now clinically available from many laboratories around the world [1]. Testing is based on the analysis of cell-free DNA in maternal plasma and involves technologies that had not previously been widely used in clinical laboratory settings. Initial proof-of-principle studies, clinical trials, and some clinical experience reports have documented high sensitivity and specificity of the testing, relative to traditional fetal aneuploidy screening [2,3].

NIPS is high-complexity, multi-component testing where the distinction between affected and unaffected cases relies on recognizing relatively minor differences in DNA patterns. It is therefore incumbent on the provider laboratories to clearly demonstrate adequate test performance both prior to launch and after the test is clinically available through ongoing quality assurance processes. In the US, laboratories are required to develop and follow written protocols that “monitor a quality system for all phases of the total testing process” [4]. The College of American Pathologists proposed that key quality indicators be regularly evaluated, including specimen identification, test order accuracy, specimen acceptability, turnaround time, reporting, and client satisfaction [5]. An ongoing retrospective assessment of the overall test performance can provide assurance that all components of testing have remained constant. Evidence for continued consistency of test performance and a description of a program to ensure quality assurance has not yet been published for NIPS.

Positive predictive values (PPV) are considered to be key reporting variables, although specific minimum values for each particular chromosome abnormality have not been defined [6]. In this report, we present summary statistical measures of test performance based on PPV and show how this can be used in quality assessment.

## 2. Materials and Methods

NIPS for trisomy 21, 18, 13 and sex chromosome abnormalities was based on evaluation of single nucleotide polymorphisms (SNPs) with testing carried out in a CLIA approved laboratory meeting College of American Pathologists requirements. The test methodology has been described elsewhere [7,8]. Testing was subject to revisions in the protocols in April 2015 (version 2), February 2016, and January 2018 (version 3) [9,10]. An algorithm to screen for a select group of microdeletions was introduced in March 2014 with procedural and algorithm changes in April 2015, February 2016, and January 2018 [10,11,12].

A quality assurance program to monitor sensitivity and specificity was established in the fourth quarter of 2013 in accordance with ISO 13485 standards. PPV was based on an expectation that the testing would perform in a manner consistent with an initial clinical experience [13]. In each quarter, a randomly selected group of approximately 200–400 test-positive cases were identified to request pregnancy outcome information. Individual referring physician offices or clinics were contacted via telephone, facsimile, or email approximately 9 to 12 months after receiving the high-risk NIPS result. Up to three attempts were made to solicit outcome data for each case. The information requested for each case included: the presence or absence of ultrasound anomalies and/or soft markers for aneuploidy, if and when diagnostic testing was pursued as well as the methodology used for testing and the test results, and the outcome of the pregnancy. Supplemental Methods 1 shows the information requested for each case. Only pregnancy outcome data requested from physician offices or clinics was used for analyses. Pregnancy outcome data was gathered and analyzed on a quarterly basis.

We used two levels of information to distinguish between a true-positive and a false-positive. First, cases with a definitive diagnosis through G-banded chromosome analysis, microarray, quantitative PCR, or MLPA (“genetic testing”) were classified as having known “truth” for PPV calculations. Second, cases with a definitive diagnosis, as described above, combined with those with presumptive evidence through ultrasound (see Supplemental Table S1 for criteria considered sufficient to classify as a true-positive) or a fetal loss were considered affected for PPV calculations. PPV was defined as (true-positives)/(true-positives + false-positives). Cases with no follow-up (either due to lack of provider response to requests, no follow-up testing, or patient transfer) were excluded from the PPV calculation.

To review whether laboratory or other changes affected test performance, decision rule tables for testing were developed to define the minimum number of confirmed true positives needed for 70% PPV non-inferiority for trisomy 21 and 18 in each quarterly reporting period [14]. A secondary set of decision rules were also developed for 80% non-inferiority as a future standard for improved performance. The approach was used to ensure a minimum acceptable PPV for a sample of representative cases. Due to lower disease prevalence and higher pregnancy loss rates without genetic testing for trisomy 13 and monosomy X, there was insufficient pregnancy outcome data for decision rule analysis on a quarterly basis. The design of the non-inferiority test is described in Supplemental Methods 2 and Supplemental Tables S2–S4. Determination of number of test-positive cases for which outcome information was needed to perform noninferiority testing was determined by the Clopper-Pearson exact binomial method, with the type 1 error rate held at level 0.05. For example, if in a given quarter, follow-up was gathered in 63 trisomy-21 test-positive cases, at least 47 would be required to be true-positives to achieve 70% of the PPV found in early experience with the test.

Summary data for years 2014–2017 were compiled. These data were used to calculate the overall observed PPV for each chromosome abnormality, with separate consideration of women aged 35, or more, and those younger than 35. Information on the use of maternal serum and ultrasound screening prior to NIPS was not routinely gathered and not taken into consideration in test reporting on the summary analyses presented here.

Referring physicians and centers were encouraged to report all instances of false-negative results to the laboratory. In suspected cases, confirmatory testing by microarray technology was available through the laboratory at no cost. Since reports of false negative cases are not based on randomly selected cases, their rate evaluation was not based on non-inferiority statistical testing. The expected rate of false-negatives was nominally set at one false negative per 10,000 cases for each specific chromosome abnormality. Because of the small numbers, we only reviewed the quarterly rate of false-negative cases for all abnormalities combined.

Collection of the data in this study was conducted as part of quality assurance standards that does not require Investigational Review Board (IRB) approval. Publication of these data was approved by an independent IRB (E&I ID 19040-01).

## 3. Results

### 3.1. Changes in The Referral Population and Positive Test Rates

**Figure 1:**
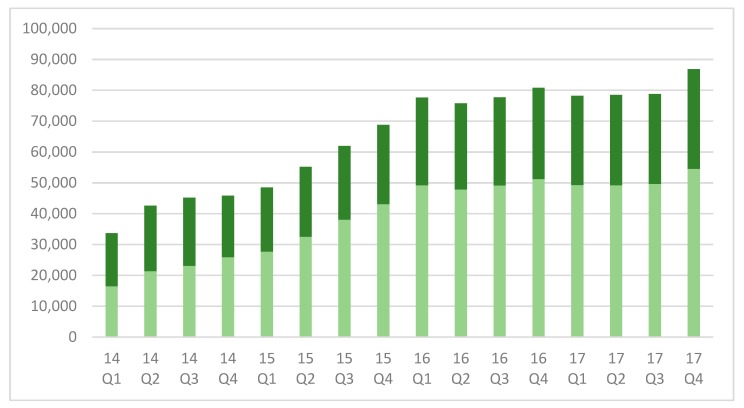
Cases reported by quarter, 2014–2017. Light green = women < 35; Dark green = women ≥ 35.

Between January 1, 2014 and December 31, 2017, a total of 1,035,844 test results were reported of which, 13,231 (1.3%) had a high-risk result for trisomies 21, 18, 13, or monosomy X. Over the 4-year period included in this analysis, there was a steady increase in test volume, increasing from 33,654 reported tests in the first quarter of 2014 to 86,799 in the fourth quarter of 2017 (Figure 1).

**Figure 2:**
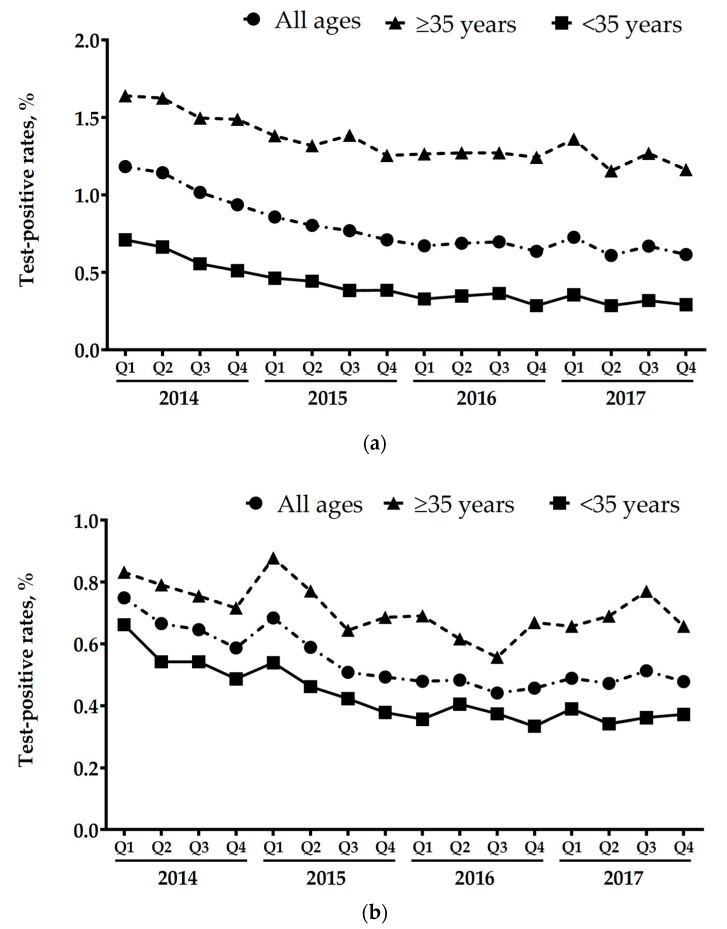
Test-positive rates by quarter, 2014–2017. (**a**) Test positive for trisomy 21; (**b**) Test positive for either trisomy 18, trisomy 13, or monosomy X.

Initially, 51% of the NIPS tests performed were from women aged greater than, or equal to 35 at their estimated date of delivery (EDD), but this declined to 37% by the end of 2015. The proportion of women 35, or more, remained approximately constant through the remainder of the study period. Consistent with a trend towards increased use of the testing by younger women, the test-positive rate significantly declined; for trisomy 21 from 398/33,654 (1.18%) to 534/86,799 (0.62%) (*p* < 0.0001) and for trisomy 18, trisomy 13 and monosomy-X combined from 252/33,654 (0.75%) to 415/86,799 (0.48%) (*p* < 0.0001). Declines in the test-positive rate were noted in both older and younger women that were suggestive of increased use of the testing by women without additional prior risk factors (Figure 2).

### 3.2. Overall Test Performance

Table 1 summarizes the overall numbers of positive tests, the numbers of samples selected for solicitation of outcomes, numbers of cases with outcome information, and the observed overall PPVs for the 4-year period.

Of those women with a positive result for fetal trisomy 21, 30.6% were aged ≤35. Consistent with a random selection of cases for solicitation of outcome information, 30.3% of those solicited and 30.5% of those with outcome information were ≤35. The corresponding proportions of women ≤35 for trisomy 18, trisomy 13 and monosomy X combined were 47.3% for all screen-positive, 45.1% for outcome solicited, and 46.0% for those with outcome information available.

The overall proportion of high-risk tests selected for follow-up was 30.8% (4071/13,231). Among these, outcome data based on genetic testing alone was available in 35.7% (1455/4071) and outcome information based on the combination of genetic testing, ultrasound or pregnancy loss was available in 50.2% (2044/4071). For cases with outcome information based on genetic testing alone, the overall PPVs for trisomies 21, 18, 13 and monosomy X were 94.7%, 91.3%, 67.8% and 77.5%, respectively. The PPVs for genetic testing, ultrasound abnormality or resulting in fetal loss information combined were 95.7%, 93.9%, 73.2%, and 87.2%, respectively. Table 1 also summarizes these data separating results for women aged <35 from those ≥35. PPVs for the younger age group were only modestly lower than that for older women despite an approximately 2.6-fold difference in the overall test-positive rates (0.79% for younger women versus 2.03% for those aged 35 or more).

### 3.3. Non-Inferiority Analysis

For each quarter between 2014 and 2017, the outcome surveillance met the 70% non-inferiority test for trisomy 21 and 18 and for the years 2016 and 2017, the results also met an 80% non-inferiority threshold for trisomy 21.

### 3.4. Trends in Positive Predictive Values

**Figure 3:**
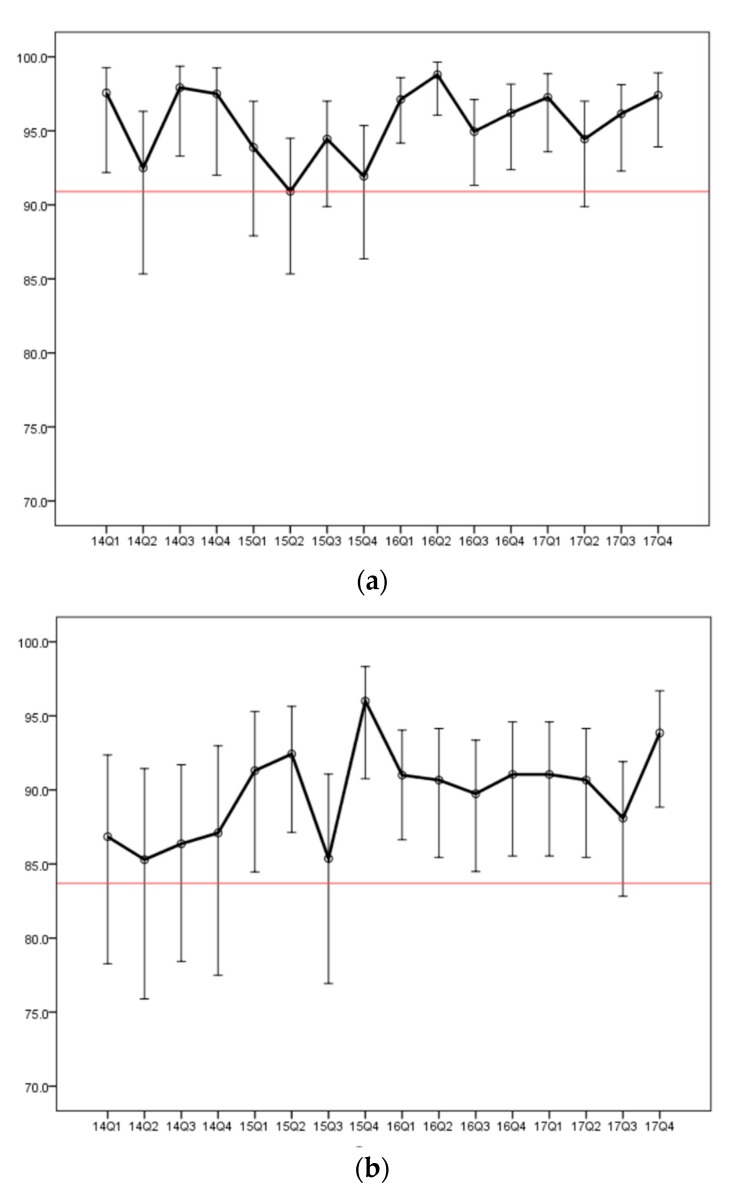
Observed positive predictive values (%) for each quarter. (**a**) trisomy 21; (**b**) trisomy 13, trisomy 18 and monosomy-X combined. True positives are based on combined evidence from follow-up with genetic testing, ultrasound findings, or fetal loss. Error bars denote 80% confidence intervals. Shorter bars at later quarters reflect larger numbers of cases used to determine PPV. Red lines denote lower boundary values (90.9% for T21 and 83.7% for T18, T13, and MX) and are based on the mean minus two standard deviations. All PPVs exceed the lower boundary values. Upper boundary values are not shown.

For the full four-year dataset, a post hoc analysis was carried out to further evaluate consistency in performance. For each quarter, for trisomy 21, for women of all ages, the PPV was based on an average of 55 cases with diagnostic genetic information. Based on these data, there would be an expectation that 80% of quarterly PPVs would have values within in the confidence interval range 89.2% to 97.3%. In 14 of 16 (87.5%) quarters the PPV exceeded the lower limit PPV of 89.2%. The results for trisomy 21 evaluated by genetic information combined with cases having presumptive ultrasound findings or pregnancy loss exceeded the lower confidence limit of 91.2% for 15 of 16 quarterly periods. Similarly, for trisomies 18, 13 and monosomy X combined, 14 of 16 quarters had a PPV exceeding the lower 80% confidence limit for data based on genetic testing (limit 74.0%). For genetic testing plus ultrasound and losses, all quarters exceeded the lower confidence limit of 83.9%. Figure 3 shows plots for the PPV values for each quarter.

Each quarter showed a PPV that exceeded a minimum boundary value of the mean minus two standard deviations.  Supplemental Figure S1 shows PPVs for each quarter for women 35 and older and for women less than 35 years.

### 3.5. False-Negative Results

The overall rate of false-negatives reported to the laboratory was 0.012% (118/1,023,042): for trisomy 21, 66 reports (1 in 15,576); trisomy 18, 33 reports (1 in 31,322); trisomy 13, 8 reports (1 in 129,330) and monosomy-X, 11 reports (1 in 93,984). The highest reported false-negative rate for all four abnormalities combined was 15/61,202 (1 in 4080) which was less than the cumulative maximum assigned nominal rate of 4 × 1 in 10,000 or 1 in 2500. There was a statistically significant trend towards a lower rate of reported false-negative results over the study period (Chi-square test for linear trend, *p* = 0.017). The rate of reported false-negative cases is shown in Supplemental Figure S2.

## 4. Discussion

In this report, we present the results of one aspect of ongoing quality assurance that was designed to provide information on the performance of a SNP-based NIPS for fetal aneuploidy. We show consistency of PPV over a 4-year testing interval.

During the four-year period, there was a shift towards increased use of the testing by younger women with a concomitant decline in positive test results, consistent with a lower a priori risk in the referral population. Theoretically, this should translate into a lower PPV because PPV is dependent on prevalence. In practice, we observed no material change in PPV over time and found only a small difference in the PPVs for younger versus older women. This may be explained by the fact that younger age may be associated with a lower false-positive rate. For example, confined placental mosaicism involving trisomy 21, trisomy 18 and trisomy 13 can originate from a meiotic cell segregation error (maternal age dependent), followed by somatic cell loss of one copy of the trisomic chromosome [15]. Previous studies that have evaluated NIPS in all-risk populations have also noted relatively small differences in the PPVs for older versus younger referral populations [16,17]. The relative stability of the PPV across populations allows this to be used as a test quality assurance measure, even in the setting of an evolving referral base.

The estimation of PPV is based on incomplete follow-up, and we cannot exclude the possibility cases with follow-up are not reflective of the full cohort. The overall follow-up collection rates in this study are comparable to those previously reported for NIPS provided by US commercial laboratories [13,18,19]. Collection of outcome information is reliant on the cooperation of referring providers and this voluntary aspect, combined with fragmented healthcare services, patient transfers, and concerns over privacy, makes achievement of high follow-up collection rates difficult. Those cases with additional testing or ultrasound information may be preferentially reported back to the laboratory or, conversely, those that result in pregnancy losses may be under-reported. There may also be increased follow-up in cases where the outcome was inconsistent with that predicted by the test. These possible biases should not materially change over time and should therefore have little impact when using change in observed PPV as a serial quality metric. Based on the proportions of women aged ≤ 35, the cases where outcomes were provided did appear to be representative of all test-positive cases.

The non-inferiority test was initially designed for a sample where at least 29 cases test-positive for trisomy 21 and/or 24 test-positive for trisomy 18 would have outcome information (Supplemental Tables S3 and S4), with a 70% non-inferiority of previously established PPVs [13]. Each laboratory considering a similar approach would need to establish its own target PPVs based on their test expectations. For laboratories with fewer test-positive cases, accumulation of cases for a longer interval could be considered.

False-negative cases may be under-reported to the laboratory and our observed trend towards declining false-positives could simply reflect a growing recognition that NIPS is imperfect and that occasionally affected pregnancies will not be detected. Without comprehensive follow-up of large numbers of negative results, a reliable estimate of the false-negative rate is not possible. From the standpoint of quality assurance monitoring, a benchmark level of reported false-negative results needs to be determined based on laboratories’ past experience.

## 5. Conclusions

The information needed to determine PPV is not available until after delivery and from a laboratory test quality perspective, departures from test performance expectations require an early response. Monitoring PPV as a quality assurance metric must therefore be regarded as only one component of laboratory oversight. Other elements include a review of the completeness of samples and test requisition information, pre-analytic assessment of equipment and reagents, maintenance of critical laboratory conditions, sample tracking, an evaluation of test-positive rates, test-failure rates, turn-around times, computer record integrity, and a survey of overall client satisfaction. Laboratory regulatory agencies play an important role in ensuring that high quality testing is maintained by all test providers.

In summary, we advocate routine evaluation of PPV by laboratories providing NIPS. This can provide an overview of past testing and helps demonstrate long-term consistency and confidence in laboratory performance. These data are also useful in the establishment of performance standards for new methodologies.

## Figures and Tables

**Table 1:** Overall PPVs for cases with follow-up information in the 4-year study interval.

	Test	All Positive (%)	Follow-Up Solicited	Confirmation by Genetics			Confirmation by Genetics, Ultrasound, or Loss		
				Follow-up Received	Abn Confirmed	PPV% (95% CI)	Follow-up Received	Abn Confirmed	PPV% (95% CI)
**All referrals** **(1,035,844)**	T21	7802 (0.75)	2347	884	837	94.7 (93.0–96.0)	1,083	1036	95.7 (94.3–96.7)
	T18	2205 (0.21)	845	333	304	91.3 (87.8–93.9)	476	447	93.9 (91.4–95.7)
	T13	1207 (0.12)	344	118	80	67.8 (58.9–75.6)	186	148	79.6 (73.2–84.7)
	MX	2017 (0.19)	535	120	93	77.5 (69.2–84.1)	299	272	91.0 (87.2–93.7)
	All	13,231 (1.28)	4071	1455	1314	90.3 (88.7–91.7)	2,044	1903	93.1 (91.9–94.1)
**Referrals from women <35** **(628,242)**	T21	2388 (0.38)	711	271	248	91.5 (87.6–94.3)	339	316	93.2 (90.0–95.4)
	T18	666 (0.11)	256	105	92	87.6 (80.0–92.6)	152	139	91.4 (85.9–94.9)
	T13	540 (0.09)	149	46	27	58.7 (44.3–71.7)	84	65	77.4 (67.4–85.0)
	MX	1361 (0.22)	372	76	59	77.6 (58.2–77.4)	212	195	92.0 (87.5–94.9)
	All	4955 (0.79)	1488	498	426	85.5 (82.2–88.4)	787	715	90.9 (88.6–92.7)
	T21	5414 (1.33)	1636	613	589	96.1 (94.2–97.4)	744	720	96.8 (95.3–97.8)
**Referrals from women ≥35** **(407,602)**	T18	1539 (0.38)	589	228	212	93.0 (88.9–95.6)	324	308	95.1 (92.1–96.9)
	T13	667 (0.16)	195	72	53	73.6 (62.4–82.4)	102	83	81.4 (72.7–87.7)
	MX	656 (0.16)	163	44	34	77.3 (63.0–87.2)	87	77	88.5 (80.1–93.6)
	All	8,276 (2.03)	2583	957	888	92.8 (91.0–94.3)	1257	1188	94.5 (93.1–95.6)

Abbreviations: Abn abnormality; T trisomy; M monosomy; PPV positive predictive value; CI confidence interval.

## Supplementary Materials

The following are available online at https://www.mdpi.com/2077-0383/8/9/1311/s1, Supplementary Methods 1: Information request form for cases selected for follow-up, Supplementary Methods 2: The design of the non-inferiority test, Table S1: Ultrasound findings considered to be sufficient for confirming a true positive trisomy, Table S2: PPVs required to meet the non-inferiority thresholds of 70% and 80% of that established for the test based on a prior publication, Table S3: Number of cases needed with follow-up and the number of true positives needed to meet non-inferiority for trisomy 21, Table S4: Number of cases needed with follow-up and the number of true positives needed to meet non-inferiority for trisomy 18, Figure. S1. Positive predictive rates by quarter, 2014–2017, Figure S2: Rate of false-negative results (trisomies 21, 18, 13 and monosomy X combined) reported to the laboratory for each quarter.
