# Supplementary material for: Quality Assurance of Non-Invasive Prenatal Screening (NIPS) for Fetal Aneuploidy Using Positive Predictive Values as Outcome Measures
Source: J Clin Med. 2019 Aug 26;8(9):1311. doi: 10.3390/jcm8091311 (PMC6780279; doi:10.3390/jcm8091311)

Supplemental Methods 1. Information request form for cases selected for follow-up

Patient Name: ______________________ **Date of Birth:** ______________________

Panorama Result: High Risk for ______________________ **Natera Case ID: received in lab on** ______________________

.

1. Were anomalies identified on ultrasound?

☐ **Yes** ☐ **No**

1a) If yes, please select the systems affected and specify anomaly:

☐ Heart:
☐ Brain:
☐ Kidney:
☐ Placenta:
☐ Pulmonary:
☐ Gastrointestinal:
☐ Increased NT:
☐ Liver:
☐ Skeletal:
☐ Facial:
☐ Growth retardation:
☐ Hands or feet:
☐ Soft markers:
☐ Other:

2. Did the patient have any of the following diagnostic tests?

☐ Amniocentesis
☐ Product of conception testing
☐ CVS
☐ Postnatal/cord blood
☐ No diagnostic testing performed

2a) If Diagnostic testing was performed, what were the results of the diagnostic testing? ☐ Not Applicable

☐ Confirmed affected ☐ Trisomy 21 ☐ Trisomy 18 ☐ Trisomy 13 ☐ Monosomy X ☐ **Other**:____________________________

Was the condition mosaic? ☐ Yes ___________% ☐ No

☐ Normal Chromosomes

2b) Which method was used to confirm this finding?

☐ FISH/RAD
☐ Karyotype
☐ Other:
☐ Microarray/CMA
☐ Physical Exam

---

3. What was the pregnancy outcome?

☐ Live birth with anomaly
☐ Live birth normal
☐ Miscarriage <14 weeks
☐ Intrauterine demise >28 weeks
☐ Neonatal death
☐ Other:
☐ Lost to follow-up
☐ Miscarriage 14-28 weeks
☐ Termination If yes, reason:
 ☐ Personal ☐ Fetal anomaly ☐ Maternal Health

In the case of a pregnancy loss, was testing performed on the products of conception? Results?

---

4. Was testing performed after the child's birth? ☐ **Yes** ☐ **No**

Was the child a male or female? ___________________

Was the child affected with a chromosome abnormality? ☐ **Yes; Diagnosis?**__________________ ☐ **No**

Additional comments or relevant information about the pregnancy:

Supplemental Methods 2. The design of the non-inferiority test.

The non-inferiority test is based on the hypothesis there is a fixed PPV for each chromosome abnormality. For a given fixed sample size of cases with follow-up, there is a minimum number of positive calls that would lead to rejection of the (null) hypothesis that the true PPV is less than either 70% or 80% of the expected PPV. Rejecting this type of null hypothesis enables one to claim that one has found statistical evidence that the true PPV is more than a chosen percentage of the target PPV. The required sample size is based on a requirement of 95% statistical power.

Expected PPVs for each chromosome abnormality were based on initial observations with the test SNP based NIPT. Due to increased pregnancy loss rates, lower disease prevalence, less robust initial estimates of the expected PPV, and sufficient pregnancy outcome data, the decision rule analysis was not applied to trisomy 13 and monosomy X. Supplemental Table 1 summarizes the target PPVs when applying the requirement of 70% or 80% PPV. Supplemental Tables 2 and 3 shows the number of cases that would need to be confirmed as true positive out of the total number with follow-up (N) to conclude that the testing was not inferior to that required.

Supplemental Table S1. Ultrasound findings considered to be sufficient for confirming a true positive trisomy

Trimester	Type*	Trisomy 21	Trisomy 18	Trisomy 13	Monosomy X
1st	Minor	Hypoplastic nasal bone	Hypoplastic nasal bone		
1st	Major	NT≥3 mm; cystic hygroma	NT≥3 mm; cystic hygroma; anencephaly; omphalocele	NT≥3 mm; holoprosencephaly; omphalocele, megacystis	NT≥3 mm; cystic hygroma
2nd	Minor	EIF; pyelectasis ≥4 mm; 10-15 mm ventriculomegaly; CPC; shortened femur/humerus; increased BPD/FL; polyhydramnios	CPC; rocker bottom feet; clenched hands	polydactyly	Thickened NF; shortened humerus/femur; fetal growth restriction
2nd	Major	NF≥7 mm; CHD; “double-bubble” sign; cystic hygroma	Fetal growth restriction; NTD; CHD; omphalocele	Midline intracranial anomaly; midline or lateral cleft lip/palate; CHD; omphalocele; clubfeet	Cystic hygroma; CHD; kidney malformation; hydrops
3rd	Minor	pyelectasis≥7 mm; + everything noted in 2nd trimester	See second trimester	See second trimester	See second trimester
3rd	Major	See second trimester	See second trimester	See second trimester	See second trimester

*At least one major, or two minor, ultrasound findings needed to be present in each case to be considered a true positive

Supplemental Table S2. PPVs required to meet the non-inferiority thresholds of 70% and 80% of that established for the test based on a prior publication.

Trisomy	Historic PPV[1]	70% of Historic PPV	80% of Historic PPV
21	90%	63%	72%
18	93%	65%	74%

[1]Historic PPV based Dar P, Curnow KJ, Gross SJ, et al. Clinical experience and follow-up with large scale single-nucleotide polymorphism-based noninvasive prenatal aneuploidy testing. Am J Obstet Gynecol. 2014 Nov;211(5):527.e1-527.e17.

Supplemental Table S3: Number of cases need with follow-up and the number of true positives needed to meet non-inferiority for trisomy 21.

	70% Noninferiority	80% Noninferiority
N	Min Confirmed Positives	Min Confirmed Positives
29	23	NA
30	24	NA
31	25	NA
32	26	NA
33	26	NA
34	27	NA
35	28	NA
36	28	NA
37	29	NA
38	30	NA
39	30	NA
40	31	NA
41	32	NA
42	33	NA
43	33	NA
44	34	NA
45	35	NA
46	35	NA
47	36	NA
48	37	NA
49	37	NA
50	38	NA
51	39	NA
52	39	NA
53	40	44
54	41	45
55	41	46
56	42	47
57	43	47
58	43	48
59	44	49

60	45	50
61	46	51
62	46	51
63	47	52
64	48	53
65	48	54
66	49	54
67	50	55
68	50	56
69	51	57
70	52	57
71	52	58
72	53	59
73	54	60
74	54	60
75	55	61
76	56	62
77	56	63
78	57	64
79	58	64
80	58	65
81	59	66
82	60	67
83	60	67
84	61	68
85	62	69
86	62	70
87	63	70
88	64	71
89	64	72
90	65	73
91	66	73
92	66	74
93	67	75
94	68	76
95	69	76
96	69	77
97	70	78
98	71	79

Supplemental Table S4: Number of cases need with follow-up and the number of true positives needed to meet non-inferiority for trisomy 18.

N	70% Noninferiority<br>Min Confirmed Positives	80% Noninferiority<br>Min Confirmed Positives
24	20	NA
25	21	NA
26	22	NA
27	23	NA
28	23	NA
29	24	NA
30	25	NA
31	25	NA
32	26	NA
33	27	NA
34	28	NA
35	28	NA
36	29	NA
37	30	NA
38	30	NA
39	31	NA
40	32	NA
41	33	NA
42	33	NA
43	34	NA
44	35	38
45	35	39
46	36	40
47	37	41
48	38	42
49	38	42
50	39	43
51	40	44
52	40	45
53	41	45
54	42	46
55	43	47

Supplemental Figure S1. Positive predictive rates by quarter, 2014-2017.

(a) Trisomy 21

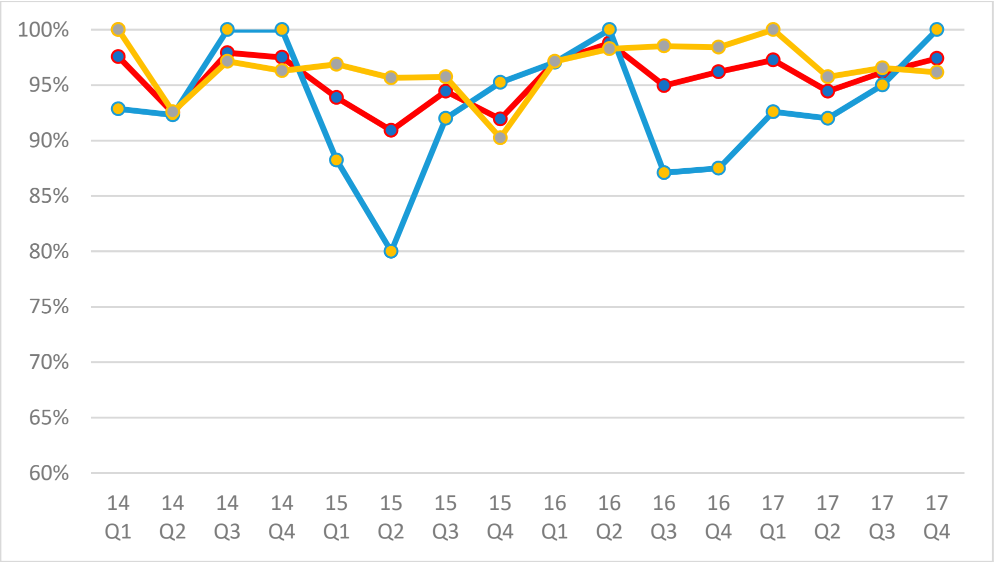

(b) Trisomy 18, trisomy 13, or monosomy X combined

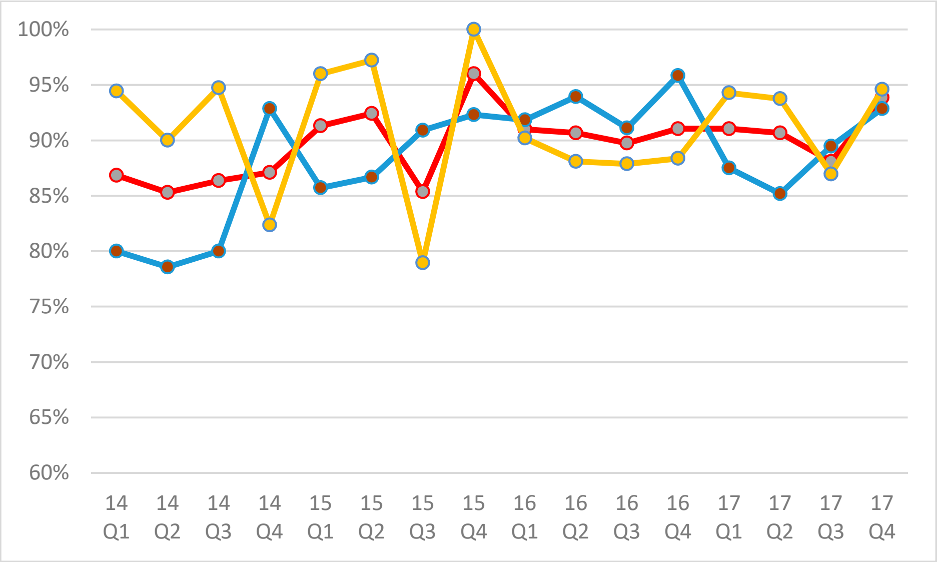

Blue = women <35; Yellow women ≥35; Red all referrals combined

Supplemental Figure S2. Rate of false-negative results (trisomies 21, 18, 13 and monosomy X combined) reported to the laboratory for each quarter.

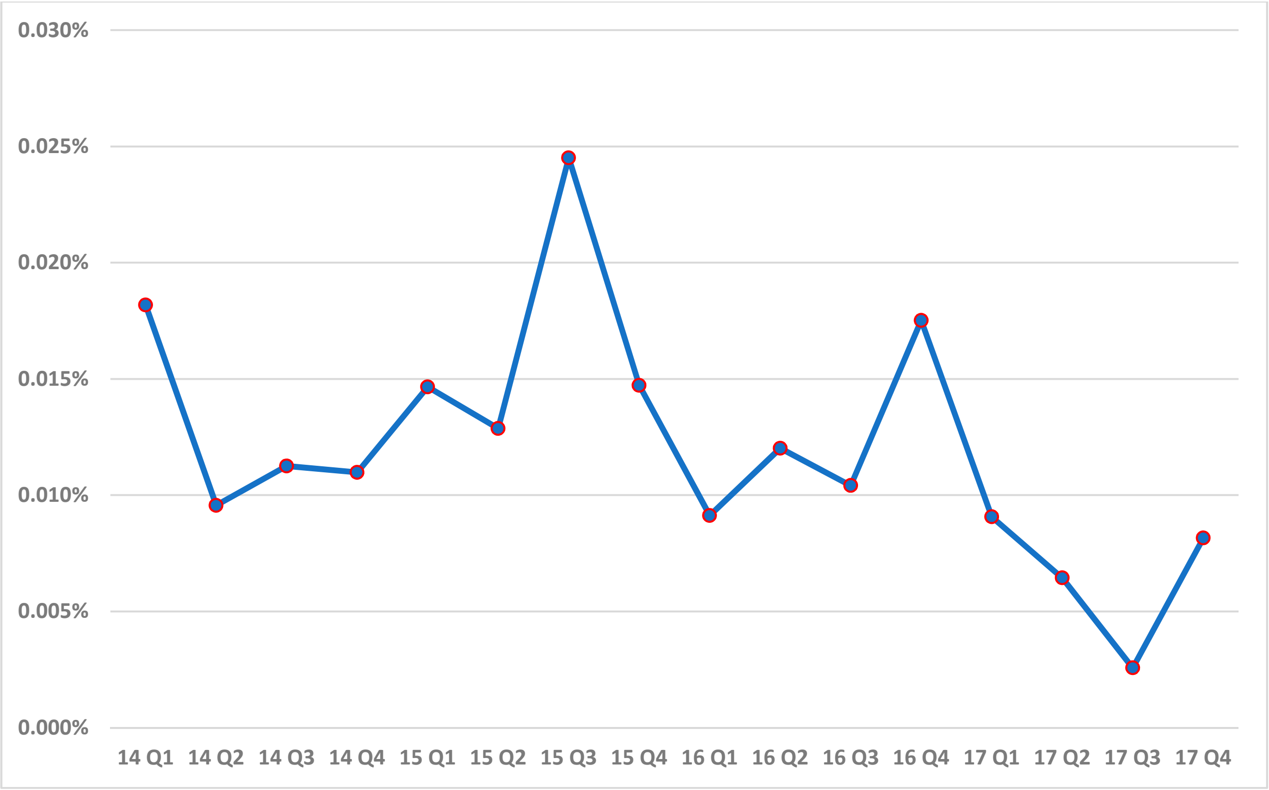

Supplement: Supplementary file 1 [file jcm-08-01311-s001.pdf]
